# Conversion Bariatric Surgery, Ketogenic Diet, and Intermittent Fasting in Bariatric Surgery Patients with Recurrent Weight Gain: a Prospective Randomized Controlled Trial

**DOI:** 10.1007/s11695-026-08654-w

**Published:** 2026-04-11

**Authors:** Seher Şen, Nihal Zekiye Erdem, Doğukan Durak

**Affiliations:** 1 Department of Nutrition and Dietetics, Faculty of Health Sciences, Mudanya University, Bursa, Turkey; 2 Department of Nutrition and Dietetics, Institute of Health Sciences, Istanbul Medipol University, Istanbul, Turkey; 3 Department of Nutrition and Dietetics, College of Health Sciences, Istanbul Medipol University, Istanbul, Turkey; 4 Department of General Surgery, Doruk Private Hospital, Bursa, Turkey

**Keywords:** Obesity, Recurrent weight gain, Conversion bariatric surgery, Ketogenic diet, Intermittent fasting

## Abstract

**Background:**

Recurrent weight gain (RWG) after metabolic and bariatric surgery (MBS) increases the need for alternative treatment strategies. This study evaluated the effects of conversion bariatric surgery (CBS), very low-calorie ketogenic diet (VLCKD), and time-restricted intermittent fasting (TRIF) on anthropometric measurements, biochemical parameters, and dietary habits in patients who experienced suboptimal clinical response (SCR) or RWG after bariatric surgery.

**Methods:**

This study included 56 patients, allocated to four groups (CBS, VLCKD, TRIF, and control; *n* = 14 each). Weight, waist-hip measurements, body composition, glycemic/lipid profile, and serum levels of specific vitamins and minerals were assessed at baseline and at week 6. Energy and nutrient intakes were calculated using BeBiS-9.

**Results:**

Data were analyzed with SPSS 22.0. The percentages of total and excess weight loss differed significantly among the groups (*p* < 0.001), with CBS (9.07–28.5%), VLCKD (9.12–31.85%), TRIF (5.09–14.97%), and control (0.97–3.40%). Additionally, the pre- and post-intervention differences in fasting insulin, HOMA-IR, HbA1c, cholesterol, LDL-C, triglyceride, and uric acid levels varied significantly among the groups. VLCKD showed a more prominent effect on glycemic parameters, whereas CBS had a more beneficial impact on the lipid profile. In intervention groups, daily energy, carbohydrate and fat intake (g/day) decreased; protein percentages increased; the frequency of consumption of energy-dense foods decreased; and healthy food preferences increased.

**Conclusions:**

Consequently, clinically significant improvements in weight management and metabolic parameters were observed in CBS, VLCKD, and TRIF groups under multidisciplinary team follow-up. These findings suggest that dietitian-led VLCKD and TRIF interventions may be considered as alternative treatment options before deciding on CBS.

**Supplementary Information:**

The online version contains supplementary material available at 10.1007/s11695-026-08654-w.

## Introduction

Obesity, defined as excessive adipose tissue accumulation that impairs health, affects one in eight individuals globally [1]. Metabolic and bariatric surgery (MBS) remains the most effective long-term treatment [2–5]; however, some patients experience suboptimal clinical response (SCR) or recurrent weight gain (RWG) [4–8], both of which may indicate conversion bariatric surgery (CBS) [4, 7, 9, 10]. Although CBS can yield %EWL ranging from 40% to 76% and improve metabolic outcomes [3, 11–13], it is associated with higher morbidity, complication rates, longer hospitalization, and micronutrient deficiencies compared with primary procedures, underscoring the need to prioritize non-surgical approaches before reoperation [14, 15].

Very low-calorie ketogenic diets (VLCKD) have gained attention as a non-surgical option for patients with SCR or RWG, demonstrating improvements in weight and metabolic parameters [2, 3, 16, 17]. However, no studies have examined the impact of intermittent fasting, widely used in obesity management, on SCR or RWG after MBS [18, 19]. Therefore, this study aimed to compare the clinical (anthropometric and biochemical) outcomes and dietary habits of patients with SCR or RWG undergoing CBS, VLCKD, or time-restricted intermittent fasting (TRIF), relative to a control group.

## Methods

### Study Design, Participant Selection, and Randomization

This randomized controlled trial was conducted between 2024 and 2025 at a private hospital in Bursa, Türkiye, and included patients who either underwent CBS or had previously undergone MBS and subsequently experienced SCR or RWG.

The inclusion criteria were as follows: patients aged 18–45 years who had undergone BS and presented with SCR within 18 months postoperatively or with RWG after achieving initial postoperative weight loss. In this study, SCR was defined as a maximum total weight loss (%TWL) of < 20% after MBS, and RWG was defined as regaining more than 30% of the initial postoperative weight loss [20].

Exclusion criteria included pregnancy or lactation; acute illness, infection, or comorbidities that could limit treatment efficacy or safety; and participation in professional athletic activity [5].

### Interventions and Study Groups

The study included a total of 56 patients, divided into four groups: CBS (*n* = 14), VLCKD (*n* = 14), TRIF (*n* = 14), and control (*n* = 14). Of the 70 patients assessed for eligibility, 10 were excluded for not meeting the inclusion criteria, and 4 declined participation by refusing to sign the informed consent form. The CBS group comprised consecutively enrolled patients undergoing conversion bariatric surgery and was not randomized. Patients allocated to the VLCKD, TRIF, and control groups had previously undergone primary MBS and experienced RWG or SCR but had not undergone CBS; these patients were randomized in a 1:1:1 ratio using computer-generated allocation (randomizer.org).

Considering that the average recommended duration of ketogenic diet interventions in the literature is approximately six weeks [2, 3], the follow-up period was standardized to six weeks for all groups to ensure methodological consistency.

### Study Approval and Ethical Considerations

All participants received both verbal and written information regarding the study procedures, and written informed consent was obtained from each patient prior to enrollment.

Institutional permission was granted by the chief medical officer of the private hospital where the study was conducted.

The study was conducted in accordance with the principles of the Declaration of Helsinki. The study protocol was reviewed and approved by a Non-Interventional Clinical Research Ethics Committee (Approval No: E-10840098-202.3.02-625; Date: January 18, 2024). The trial was prospectively registered at ClinicalTrials.gov (Identifier: NCT06963437).

### Dietary Interventions

The nutritional management principles for all patients were established in consultation with a multidisciplinary MBS team and were designed in accordance with the recommendations of the American Society for Metabolic and Bariatric Surgery (ASMBS) guidelines. The dietary intervention protocols applied to each study group are summarized in Table 1 [2, 3, 6, 21–24].

**Table 1:** Nutritional interventions applied to the study groups

	CBS Group	VLCKD Group	TRIF Group	Control Group
Diet Characteristics	Four-phase diet program: clear liquid, full liquid, puree, and solid diet	High-protein VLCKD	16:8 IF regimen	*N/A*
Energy	N/A	600–800 kcal	N/A	N/A
Protein	23–27% 0.8–1.2 g/kg IBW/day 35 g/day Hellobari® ProteColl whey protein & collagen**	40–45% 0.8–1.2 g/kg IBW/day 35 g/day Hellobari® ProteColl whey protein & collagen**	23–27% 0.8–1.2 g/kg IBW/day	N/A
Carbohydrate	45–55%	10% (< 30 g/day CHO from vegetables)	45–55%	N/A
Fat	20–30%	40–50% (natural sources, oilseeds, and 10 g olive oil)	20–30%	N/A
Caffeine	300 mg	300 mg	300 mg	N/A

^*^N/A: not applicable

^**^Hellobari® ProteColl Whey protein&collagen, İstanbul, Turkey

### Study Outcomes and Measurements

Anthropometric and biochemical parameters were assessed at baseline (pre-intervention) and at week 6. The evaluated anthropometric variables included body weight, body mass index (BMI), waist circumference, hip circumference, waist-to-hip ratio (WHR), waist-to-height ratio (WHtR), body composition, percentage of total weight loss (%TWL), and percentage of excess weight loss (%EWL).

Weight-loss outcomes were expressed as percentage of total weight loss (%TWL) and percentage of excess weight loss (%EWL). %TWL was considered the primary outcome measure, as it provides a standardized and reproducible metric independent of ideal body weight definitions [14, 22]. %EWL, as the historically predominant metric in the bariatric literature, was reported to ensure comparability with previous studies.

  

$$\begin{aligned}&\mathrm{Percentage\;of\;total\;weight\;loss}\left({\%}\;\mathrm{TWL}\right)\\&=\frac{(\mathrm{Preoperative\; weight}-\mathrm{Postoperative\;weight})}{\mathrm{Preoperative\;weight}}\times\:100\end{aligned}$$

$$\begin{aligned}&\mathrm{Percentage\;of\;excess\;weight\; loss}\left({\%}\;\mathrm{EWL}\right)\\&=\frac{(\mathrm{Preoperative\;weight}-\mathrm{Postoperative\;weight})}{(\mathrm{Preoperative\;weight}-\mathrm{Ideal\;weight})}\times\:100\end{aligned}$$

Ideal body weight was calculated based on a BMI of 25 kg/m² [25].

Body composition was measured using bioelectrical impedance analysis (BIA) (Tanita MC-780 MA, Japan) in accordance with ESPEN guidelines [26]. All measurements were performed under standardized conditions, with participants wearing light clothing and barefoot. To ensure measurement accuracy, participants were instructed to refrain from eating for at least 4 h prior to testing and to limit water intake to ≤ 200 mL. They were also advised to avoid caffeinated beverages, alcohol, and exercise for at least 8 h before assessment. Before each analysis, all metallic items (e.g., jewelry and watches) were removed [27].

At baseline and at week 6, individual face-to-face interviews were conducted to collect dietary intake frequency data and three-day food records (including one weekend day). Participants’ daily macro- and micronutrient intakes were analyzed using a computer-assisted nutrition analysis program (Nutrition Information Systems–BEBIS 9, Version 9.0, Istanbul, Turkey) [28].

### Assessment of Ketosis

**Fig. 1:**
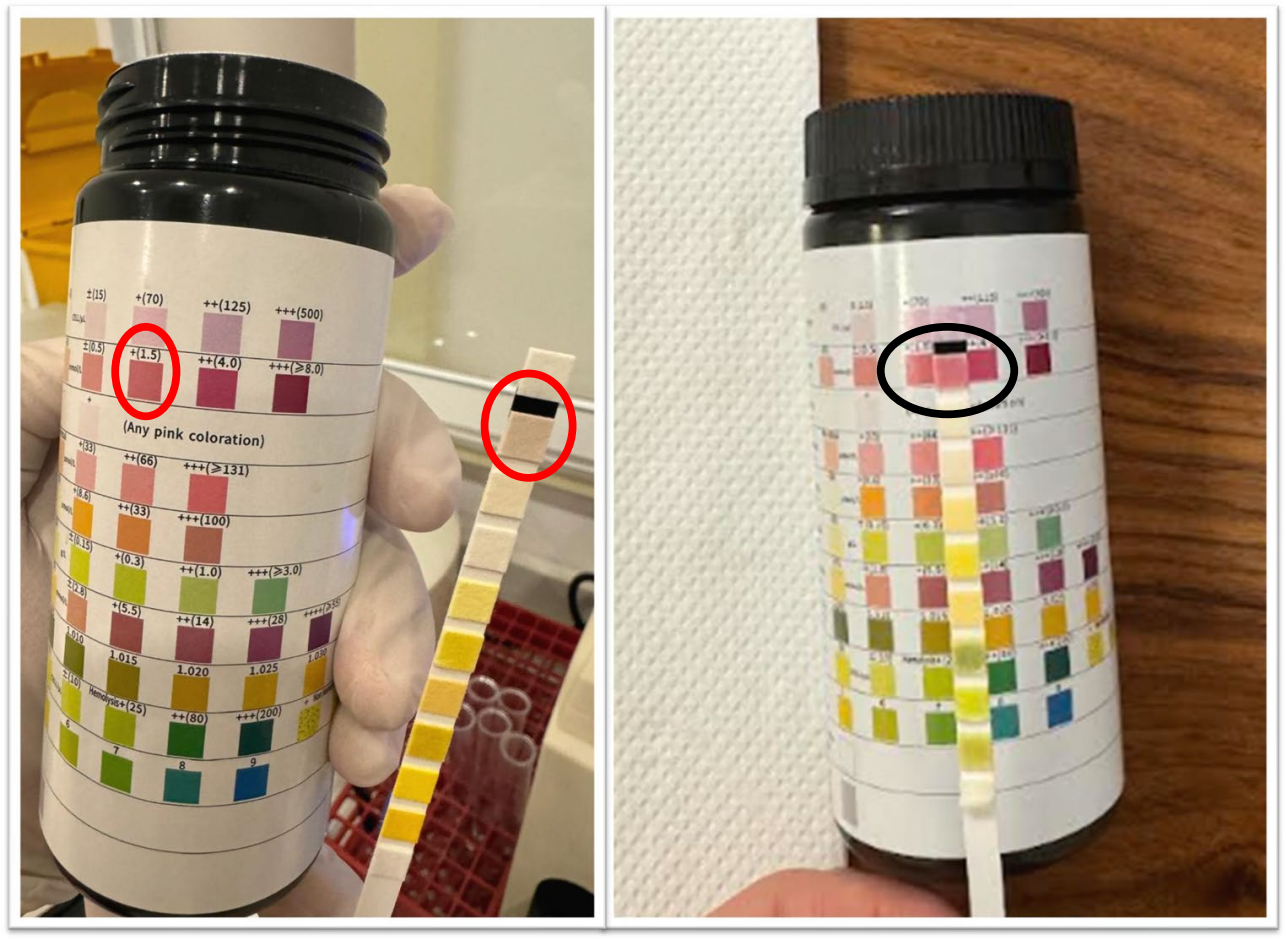
Urine ketone test strip used for the assessment of ketosis

To monitor whether participants remained in a state of ketosis, urinary acetoacetate concentrations were measured once weekly in the morning, before breakfast, using urine ketone test strips. In accordance with previous studies, a ketone value of ≥ + 1.5 mmol/L detected on the strip was considered positive for ketosis (Fig. 1) [29–34].

### Surgical Procedure

In the CBS group, the primary procedure for all patients was sleeve gastrectomy (SG). Patients were scheduled for revision surgery due to weight regain. Roux-en-Y gastric bypass (RYGB) was recommended as the revisional procedure for all patients. For the gastric bypass, a gastric pouch of no more than 30 mL was created. A jejunal loop was identified 80 cm distal to the ligament of Treitz, and a gastrojejunostomy was performed at this level. The jejunum was then measured 120 cm distal to the gastrojejunostomy, and a jejunojejunostomy was performed to create the biliopancreatic limb. All anastomoses were constructed as side-to-side anastomoses using a 45-mm laparoscopic purple linear stapler. The anastomotic defects were closed in two layers with 3 − 0 PDS sutures.

### Statistical Analysis

Normality was assessed using the Shapiro–Wilk test. As the data were not normally distributed, non-parametric tests were applied. The Mann–Whitney U test was used for comparisons between two independent groups. McNemar (2 × 2) and Bowker (R×C) tests were used to evaluate dependent categorical data. For comparisons among more than two groups, the Kruskal–Wallis test with Dunn–Bonferroni post hoc analysis was applied. Statistical analyses were performed using IBM SPSS Statistics version 27.0. A p-value < 0.05 was considered statistically significant.

## Results

**Fig. 2:**
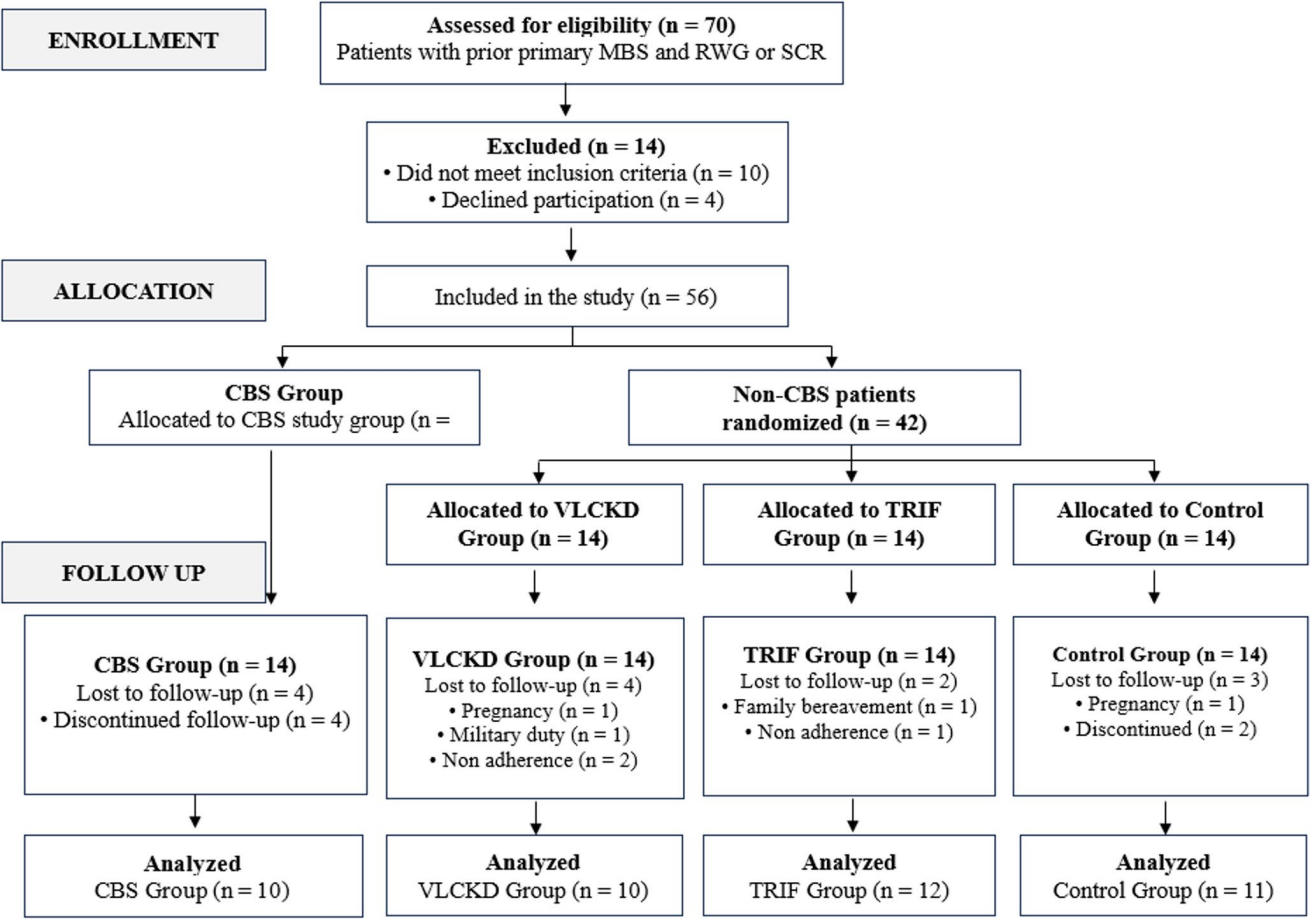
CONSORT 2010 flow diagram of participant enrollment, allocation, follow-up, and analysis. Patients undergoing conversion bariatric surgery were allocated to the CBS group, whereas patients not undergoing conversion bariatric surgery were randomized into VLCKD, TRIF, and control groups

A total of 70 patients were assessed for eligibility. Fourteen patients were excluded (10 did not meet the inclusion criteria and 4 declined participation). Fifty-six patients were included in the study. Of these, 14 underwent conversion bariatric surgery, while 42 were randomized to the VLCKD, TRIF, or control groups. During follow-up, 13 patients were lost to follow-up, and 43 patients completed the study (Fig. 2).

**Fig. 3:**
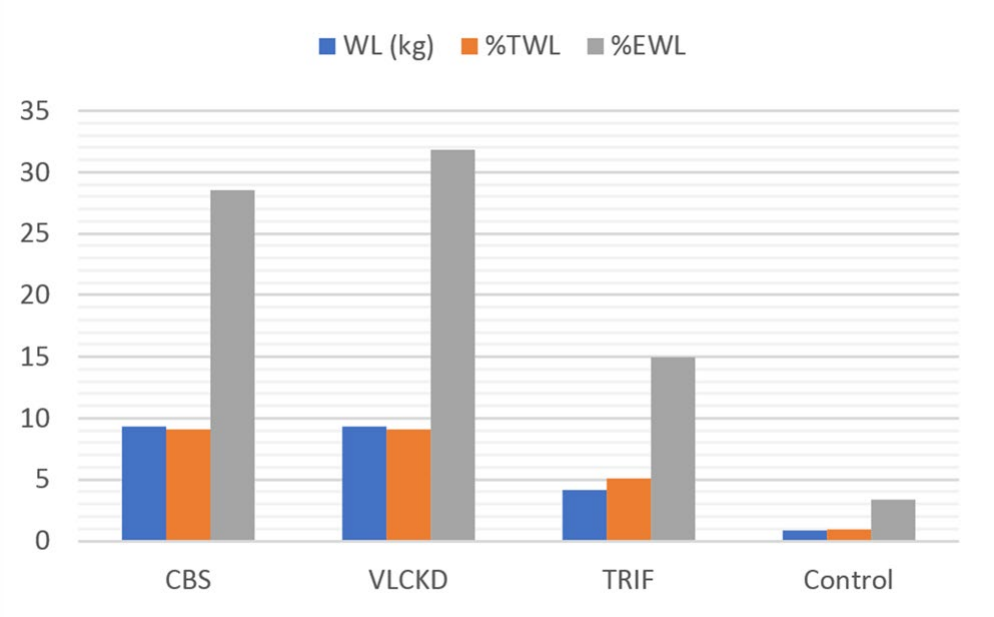
Weight loss, total weight loss percentage, and excess weight loss percentage across the study groups. WL: Weight loss; %TWL: Percentage of total weight loss; %EWL: Percentage of excess weight loss.p: Kruskal–Wallis H test. Between-group differences in WL, %TWL, and %EWL were significant (p < 0.001)

Of the 43 patients who completed the study, 37 were women (86%) and 6 were men (14%). The mean age was 38.10 ± 8.08 years. Baseline demographic characteristics are presented in Table 2. Weight-loss outcomes are shown in Fig. 3. Anthropometric changes, biochemical findings, and macro- and micronutrient intakes before and after the interventions are presented in Tables 3 and 4, and 5, respectively.

**Table 2:** Baseline Demographic Characteristics of the Patients

		CBS		VLCKD		TRIF		Control		Total	
		*n*	%	*n*	%	*n*	%	*n*	%	*n*	%
Sex	Female	9	90	8	80	11	91.7	9	81.8	37	86
	Male	1	10	2	20	1	8.3	2	18.2	6	14
Primary BS Procedure	SG	10	100	9	90	11	91.7	11	100	41	95.3
	RYGB	0	0	1	10	1	8.3	0	0	2	4.7
Insulin Resistance	Present	1	10	1	10	1	8.3	3	27.3	6	14
	Absent	9	90	9	90	11	91.7	8	72.7	37	86
T2DM	Present	1	10	1	10	0	0	0	0	2	4.7
	Absent	9	90	9	90	12	100	11	100	41	95.3
Cardiovascular Disease	Present	1	10	1	10	0	0	1	9.1	3	7
	Absent	9	90	9	90	12	100	10	90.9	40	93
Hyperlipidemia	Present	1	10	0	0	0	0	0	0	1	2.3
	Absent	9	90	10	100	12	100	11	100	42	97.7
Hypertension	Present	1	10	2	20	2	16.7	2	18.2	7	16.3
	Absent	9	90	8	80	10	83.3	9	81.8	36	83.7
GERD	Present	4	40	3	30	4	33.3	4	36.4	15	34.9
	Absent	6	60	7	70	8	66.7	7	63.6	28	65.1

*CBS* Conversion Bariatric Surgery, *VLCKD* Very-Low-Calorie Ketogenic Diet, *TRIF* Time-Restricted Intermittent Fasting, *SG* Sleeve Gastrectomy, *RYGB* Roux-en-Y Gastric Bypass, *T2DM* Type 2 Diabetes Mellitus, *GERD* Gastroesophageal Reflux Disease

**Table 3:** Comparison of Pre- and Post-Intervention Anthropometric Measurements and Within-Group and Between-Group Change

		CBS		VLCKD		TRIF		Control		
		Mean ± S.S	p*	Mean ± S.S	p*	Mean ± S.S	p*	Mean ± S.S	p*	p**
Weight (kg)	Pre	102.08 ± 21.938	**0.005**	104.62 ± 21.807	**0.005**	94.06 ± 10.312	**0.002**	97.44 ± 11.163	0.068	0.745
	Post	92.75 ± 19.947		95.41 ± 19.678		90.06 ± 9.710		96.79 ± 10.982		0.709
	Δ	9.33 ± 2.98		9.31 ± 2.62		4.13 ± 2.12		0.85 ± 0.80		**< 0.001**
BMI (kg/m²)	Pre	38.34 ± 5.605	**0.008**	37.43 ± 7.178	**0.005**	36.56 ± 3.866	**0.002**	36.02 ± 5.155	0.06	0.468
	Post	35.25 ± 5.336		34.14 ± 6.526		35.06 ± 3.850		35.77 ± 5.129		0.718
WC (cm)	Pre	114.90 ± 17.019	**0.047**	116.80 ± 21.888	**0.014**	109.92 ± 10.791	**0.002**	110.45 ± 11.449	1	0.73
	Post	106.70 ± 17.211		106.50 ± 17.329		103.17 ± 12.342		110.36 ± 10.661		0.85
	Δ	-8.20 ± 8.57		-10.30 ± 7.90		-6.75 ± 5.08		-0.09 ± 6.17		**< 0.001**
HC (cm)	Pre	128.80 ± 12.639	**0.021**	125.30 ± 13.417	**0.005**	123.92 ± 7.645	**0.002**	121.73 ± 9.572	1	0.508
	Post	121.60 ± 12.140		117.00 ± 11.576		118.50 ± 6.856		122.45 ± 9.585		0.347
	Δ	-7.20 ± 5.94		-8.30 ± 2.87		-5.42 ± 3.34		0.73 ± 3.47		**< 0.001**
WHR	Pre	0.89 ± 0.072	0.33	0.92 ± 0.108	0.092	0.89 ± 0.104	0.232	0.91 ± 0.105	0.916	0.702
	Post	0.87 ± 0.063		0.90 ± 0.082		0.87 ± 0.095		0.90 ± 0.109		0.574
	Δ	-0.02 ± 0.06		-0.02 ± 0.05		-0.02 ± 0.05		-0.01 ± 0.07		0.255
WHtR	Pre	0.70 ± 0.084	**0.041**	0.74 ± 0.159	**0.014**	0.68 ± 0.067	**0.002**	0.67 ± 0.082	1	0.199
	Post	0.65 ± 0.096		0.63 ± 0.102		0.63 ± 0.079		0.67 ± 0.081		0.257
	Δ	-0.05 ± 0.05		-0.11 ± 0.12		-0.05 ± 0.03		-0.00 ± 0.04		**0.001**
MUAC (cm)	Pre	38.65 ± 4.679	**0.008**	36.60 ± 6.883	**0.011**	34.88 ± 2.861	**0.007**	35.32 ± 3.288	0.785	0.509
	Post	35.70 ± 4.057		34.65 ± 6.263		33.54 ± 3.215		35.36 ± 3.107		0.522
	Δ	-2.95 ± 2.22		-1.95 ± 1.38		-1.33 ± 1.29		0.05 ± 0.47		**< 0.001**
NC (cm)	Pre	37.75 ± 4.480	**0.005**	37.55 ± 3.594	**0.004**	35.67 ± 2.508	**0.011**	35.91 ± 3.056	0.285	0.371
	Post	35.50 ± 4.069		35.55 ± 3.041		34.79 ± 2.491		36.14 ± 2.984		0.637
	Δ	-2.25 ± 1.11		-2.00 ± 0.94		-0.88 ± 0.98		0.23 ± 0.82		**< 0.001**
FM (kg)	Pre	44.14 ± 12.412	**0.005**	43.15 ± 15.188	**0.007**	39.79 ± 6.591	**0.002**	39.36 ± 8.063	0.286	0.404
	Post	37.76 ± 11.740		37.09 ± 13.253		36.91 ± 6.148		40.67 ± 7.831		0.375
	Δ	-6.38 ± 2.39		-6.06 ± 3.79		-2.88 ± 1.68		1.32 ± 3.22		**< 0.001**
MM (kg)	Pre	54.57 ± 9.975	**0.007**	58.34 ± 10.851	**0.012**	51.28 ± 5.666	0.065	55.88 ± 7.951	0.059	0.446
	Post	50.91 ± 9.800		56.19 ± 10.033		50.64 ± 5.283		54.17 ± 8.234		0.646
	Δ	-3.66 ± 2.26		-2.15 ± 1.74		-0.64 ± 1.08		-1.71 ± 3.89		**0.003**

*BMI* Body Mass Index, *WC* Waist Circumference, *HC* Hip Circumference, *WHR* Waist-to-Hip Ratio, *WHtR* Waist-to-Height Ratio, *MUAC* Mid-Upper Arm Circumference, *NC* Neck Circumference, *FM* Fat Mass, *MM* Muscle Mass, *Pre* Pre-intervention value, *Post* Post-intervention value, *Δ (Difference) *Post–Pre change

p* values represent within-group comparisons. p** values represent between-group comparisons

**Table 4:** Comparison of Pre- and Post-Intervention Biochemical Findings Within and Between Groups

		CBS		VLCKD		TRIF		Control		
		Mean ± S.S	p*	Mean ± S.S	p*	Mean ± S.S	p*	Mean ± S.S	p*	p**
Fasting Glucose (mg/dL)	Pre	93.70 ± 9.007	0.102	96.28 ± 16.168	**0.005**	89.75 ± 5.496	**0.007**	92.36 ± 5.006	0.262	0.441
	Post	89.78 ± 5.853		88.33 ± 8.532		88.50 ± 4.421		89.78 ± 7.156		0.646
	Δ	-3.92 ± 6.95		-7.95 ± 9.45		-1.25 ± 3.25		-2.58 ± 6.62		0.217
Fasting Insulin (µIU/mL)	Pre	10.92 ± 2.972	**0.005**	10.75 ± 5.180	**0.009**	11.51 ± 7.522	0.165	9.29 ± 1.883	0.531	0.728
	Post	7.19 ± 1.344		5.49 ± 1.307		7.28 ± 3.626		9.03 ± 2.086		**0.007**
	Δ	-3.73 ± 2.26		-5.26 ± 4.03		-4.23 ± 4.67		-0.27 ± 0.71		**< 0.001**
Homa-IR	Pre	2.51 ± 0.712	**0.005**	2.69 ± 1.943	0.767	2.58 ± 1.805	0.959	2.12 ± 0.434	0.285	0.515
	Post	1.60 ± 0.359		1.21 ± 0.416		1.59 ± 0.849		2.02 ± 0.558		**0.015**
	Δ	-0.91 ± 0.48		-1.48 ± 1.55		-0.99 ± 1.10		-0.11 ± 0.26		**0.001**
HbA1c (%)	Pre	5.85 ± 0.218	**0.005**	5.60 ± 0.362	**0.008**	5.43 ± 0.311	0.14	5.40 ± 0.329	0.104	**0.009**
	Post	5.43 ± 0.291		4.96 ± 0.234		5.26 ± 0.235		5.30 ± 0.361		**0.005**
	Δ	-0.42 ± 0.23		-0.64 ± 0.37		-0.16 ± 0.29		-0.10 ± 0.18		**< 0.001**
HDL-C (mg/dL)	Pre	49.03 ± 13.355	**0.021**	51.77 ± 14.739	0.285	59.92 ± 10.220	0.27	56.56 ± 18.165	**0.012**	0.115
	Post	41.85 ± 12.459		52.14 ± 13.311		59.25 ± 9.166		59.19 ± 17.228		**0.01**
	Δ	-7.18 ± 8.91		0.37 ± 11.10		-0.67 ± 5.69		2.64 ± 6.38		0.089
LDL-C (mg/dL)	Pre	127.47 ± 26.96	**0.009**	127.42 ± 22.761	**0.017**	111.37 ± 28.779	**0.01**	133.39 ± 25.441	0.715	0.276
	Post	102.05 ± 26.84		121.20 ± 30.499		89.83 ± 32.370		132.18 ± 28.983		**0.006**
	Δ	-25.42 ± 17.29		-6.22 ± 15.33		-21.53 ± 33.36		-1.21 ± 9.42		0.008
Total Cholesterol (mg/dL)	Pre	210.02 ± 37.07	**0.005**	202.55 ± 28.870	0.214	191.48 ± 27.213	**0.011**	210.85 ± 22.010	0.465	0.408
	Post	166.10 ± 25.93		187.00 ± 34.521		166.52 ± 38.103		211.66 ± 28.868		**0.005**
	Δ	-43.92 ± 27.74		-15.55 ± 15.53		-24.97 ± 32.20		3.70 ± 12.79		<0.001
Triglycerides (mg/dL)	Pre	138.10 ± 54.52	**0.015**	122.38 ± 50.424	0.799	102.17 ± 34.064	0.091	103.35 ± 32.252	0.109	0.19
	Post	110.40 ± 37.63		96.10 ± 30.701		87.00 ± 25.384		104.98 ± 42.561		0.469
	Δ	-27.70 ± 25.84		-26.28 ± 29.33		-15.17 ± 15.00		1.64 ± 29.78		0.017
ALT (U/L)	Pre	24.50 ± 20.657	0.093	20.70 ± 21.929	**0.008**	20.42 ± 17.789	**0.028**	12.89 ± 6.721	0.054	0.1
	Post	20.30 ± 14.080		21.15 ± 21.672		15.08 ± 5.583		12.66 ± 6.781		0.139
	Δ	-4.20 ± 7.70		0.45 ± 4.69		-5.33 ± 14.32		-0.23 ± 2.70		0.278
AST (U/L)	Pre	20.90 ± 6.871	0.624	16.60 ± 7.735	0.273	20.42 ± 11.626	0.875	14.98 ± 3.157	0.116	0.059
	Post	19.90 ± 4.771		18.61 ± 7.927		16.58 ± 3.895		15.53 ± 3.108		0.195
	Δ	-1.00 ± 4.42		2.01 ± 4.21		-3.83 ± 9.15		0.55 ± 1.86		0.075
BUN (mg/dL)	Pre	12.55 ± 4.129	0.302	18.32 ± 7.146	0.144	19.53 ± 4.520	0.126	23.01 ± 8.544	0.225	**0.002**
	Post	12.30 ± 4.191		16.39 ± 4.446		16.15 ± 3.018		19.36 ± 6.501		**0.022**
	Δ	-0.25 ± 2.42		-1.93 ± 4.57		-3.38 ± 2.35		-3.65 ± 6.02		0.093
Uric Acid (mg/dL)	Pre	5.74 ± 1.991	**0.007**	5.92 ± 1.791	**0.014**	4.68 ± 1.589	0.766	5.06 ± 1.116	**0.024**	0.156
	Post	3.73 ± 0.368		4.84 ± 0.902		4.27 ± 1.021		4.86 ± 1.052		**0.037**
	Δ	-2.01 ± 1.99		-1.08 ± 1.16		-0.41 ± 0.98		-0.20 ± 0.42		**0.009**
Urea (mg/dL)	Pre	22.00 ± 4.447	**0.005**	27.45 ± 7.272	**0.015**	21.36 ± 1.948	**0.022**	26.16 ± 7.441	0.216	**0.018**
	Post	14.90 ± 3.071		25.51 ± 10.547		20.17 ± 2.657		22.46 ± 6.664		**0.001**
	Δ	-7.10 ± 4.48		-1.94 ± 9.97		-1.19 ± 3.39		-3.70 ± 4.71		0.051
Albumin (g/dL)	Pre	42.95 ± 3.722	0.953	45.26 ± 2.761	**0.005**	42.52 ± 1.834	**0.01**	42.85 ± 2.611	0.185	0.119
	Post	43.14 ± 2.609		44.69 ± 1.602		42.34 ± 1.964		42.67 ± 2.016		**0.043**
	Δ	0.19 ± 1.94		-0.57 ± 1.98		-0.18 ± 1.97		-0.17 ± 2.19		0.955
Total Protein (g/L)	Pre	69.96 ± 6.369	**0.015**	73.92 ± 4.729	0.114	69.50 ± 4.232	0.06	68.61 ± 4.532	0.858	0.106
	Post	65.96 ± 6.465		74.48 ± 3.960		66.67 ± 2.309		62.89 ± 18.612		**0.001**
	Δ	-4.00 ± 4.46		0.56 ± 4.54		-2.83 ± 2.44		-5.72 ± 18.01		**0.008**

*HDL-C* High-Density Lipoprotein Cholesterol, *LDL-C* Low-Density Lipoprotein Cholesterol, *HOMA-IR* Homeostatic Model Assessment of Insulin Resistance, *ALT* Alanine Aminotransferase, *AST* Aspartate Aminotransferase, *BUN* Blood Urea Nitrogen, *Pre* Pre-intervention value, *Post* Post-intervention value, *Δ (Difference) *Post–Pre change

p* values represent within-group comparisons. p** values represent between-group comparisons

**Table 5:** Comparison of Pre- and Post-Intervention Daily Macro- and Micronutrient Intakes Within and Between Groups

		CBS		VLCKD		TRIF		Control		
		Mean ± S.S	p*	Mean ± S.S	p*	Mean ± S.S	p*	Mean ± S.S	p*	p**
Energy (kcal)	Pre	1530.1 ± 489.1	**0.005**	1557.1 ± 397.32	**0.005**	1476.99 ± 285.7	**0.002**	1934.66 ± 456.5	**0.021**	0.097
	Post	692.9 ± 152.88		740 ± 80.520		1053.4 ± 242.51		1812.5 ± 397.59		**< 0.001**
Protein (g)	Pre	61.73 ± 16.037	**0.007**	59.98 ± 17.256	**0.009**	53.12 ± 11.936	0.272	65.45 ± 8.075	**0.004**	0.176
	Post	57.03 ± 16.165		71.61 ± 10.805		50.42 ± 11.524		74.47 ± 12.771		**< 0.001**
Protein %	Pre	16.90 ± 2.331	**0.005**	15.50 ± 1.581	**0.005**	14.92 ± 2.466	**0.003**	14.32 ± 2.273	**0.005**	0.111
	Post	33.90 ± 5.705		39.90 ± 4.175		19.83 ± 2.823		16.97 ± 1.862		**< 0.001**
CHO (g)	Pre	155.91 ± 56.2	**0.005**	143.10 ± 54.258	**0.005**	169.91 ± 51.277	**0.002**	218.52 ± 69.816	0.013	0.191
	Post	40.62 ± 7.579		14.52 ± 2.345		83.08 ± 33.192		182.26 ± 69.210		**< 0.001**
CHO%	Pre	41.40 ± 4.477	**0.005**	37.70 ± 10.350	**0.005**	46.25 ± 7.300	**0.002**	44.29 ± 4.262	0.013	0.056
	Post	24.80 ± 4.709		8.00 ± 0.817		31.42 ± 6.680		39.17 ± 6.607		**< 0.001**
Fiber (g)	Pre	11.04 ± 2.401	**0.005**	12.38 ± 2.149	**0.005**	14.27 ± 4.062	0.06	20.27 ± 5.621	0.11	**< 0.001**
	Post	4.96 ± 1.525		7.02 ± 1.486		11.69 ± 3.775		22.09 ± 6.056		**< 0.001**
Fat (g)	Pre	71.85 ± 24.921	**0.005**	70.00 ± 14.676	**0.005**	63.24 ± 10.866	0.084	85.61 ± 18.920	0.722	0.609
	Post	32.09 ± 8.489		42.72 ± 6.223		56.19 ± 9.569		85.13 ± 12.543		**< 0.001**
Fat %	Pre	41.60 ± 3.893	0.944	40.70 ± 5.144	**0.005**	38.75 ± 5.739	**0.003**	39.27 ± 1.966	0.041	**0.039**
	Post	41.30 ± 2.983		51.90 ± 4.701		48.58 ± 5.468		42.46 ± 5.160		**< 0.001**

*CHO: Carbohydrate Pre: Pre-intervention value; Post: Post-intervention value

P* values represent within-group comparisons. P** values represent between-group comparisons

The Bowker test was used to compare pre- and post-intervention distributions of multi-categorical dietary habit variables within each group. Detailed results are provided in Supplementary Tables 1–4. As shown in these tables, consumption of milk, yogurt, fruit, eggs, meat/poultry/fish, vegetables, and salad increased by week 6, whereas intake of bread, refined grains, pastries, butter/margarine, high-fat snacks, sugary beverages, fruit juices, sweets, chocolate, biscuits, and crackers decreased.

## Discussion

To the best of our knowledge, no previous study has directly compared the effectiveness of CBS, VLCKD, and TRIF in patients who developed SCR or RWG following MBS. This critical gap in the literature underscores the originality and clinical relevance of the present trial, as it provides the first head-to-head evaluation of these distinct nutritional strategies in a population with limited evidence-based management options.

### Effect of CBS on Clinical Outcomes

Previous studies have shown that restrictive surgical procedures, particularly SG, may be associated with long-term complications such as SCR or RWG [35–37]. In our study, the finding that all but two patients had undergone SG as their primary bariatric procedure is consistent with these reports. Among CBS procedures, RYGB is one of the most commonly preferred techniques in clinical practice, as it reduces the nutritional complications that may arise from malabsorptive procedures [38, 39]. Accordingly, RYGB was the CBS procedure performed in our study.

In our study, patients who underwent CBS demonstrated significant short-term weight reduction (− 9.33 kg) during the 6-week follow-up period (*p* = 0.005). Similar findings have been reported in the literature; the weight loss observed at 6 weeks postoperatively in our study corresponds to results reported at 1 year postoperatively [40–42]. In two studies evaluating patients who underwent RYGB as a revisional procedure after SG, the mean %EWL at 1 year postoperatively was approximately 40% [21, 43]. The %EWL of 28.53% achieved in our study can therefore be considered encouraging when compared with these longer-term results. However, a systematic review noted that %EWL after CBS decreased from 19.3% at 6 months postoperatively to 10.3% at 24 months [44], suggesting that the initial weight loss achieved in the early postoperative period may diminish over time.

The reduction in energy intake, along with hormonal, anatomical, and physiological changes following RYGB, significantly lowers glucose levels and improves lipid profiles [45]. In our study, decreases in HbA1c, fasting glucose, fasting insulin, LDL-C, triglycerides, and total cholesterol indicate the positive effects of CBS on both glycemic control and dyslipidemia. These findings are consistent with those reported in previous studies [40, 45–49].

### Effect of VLCKD on Clinical Outcomes

In the literature, evidence on the use of VLCKD for RWG after bariatric surgery is limited to one case report and four clinical studies [2, 3, 17, 50]. In our study, the anthropometric improvements observed in the VLCKD group were consistent with findings reported by Vinciguerra et al. [3], Correa et al. [2], and Ernesti et al. [17], supporting the effectiveness of VLCKD in this population. By restricting carbohydrate intake to < 30 g/day, VLCKD induces nutritional ketosis, reduces glucose and insulin levels, and enhances insulin sensitivity [51]. Accordingly, we observed significant reductions in fasting glucose, insulin, and HbA1c, in line with previous reports [2, 3, 52]. Triglyceride levels also decreased, consistent with prior studies [3, 52], likely due to reduced hepatic TG synthesis and increased fat oxidation.

Although VLCKD may increase LDL-C, particularly in genetically predisposed individuals [53], our protocol limited saturated fats and permitted only olive oil, similar to other studies [3, 17, 52]. As a result, LDL-C did not increase; instead, a modest but significant reduction (− 6.22 mg/dL, *p* = 0.017) was observed, comparable to Ernesti et al. [17], although smaller than the decrease reported by Vinciguerra et al. [3]. These differences may reflect variations in baseline metabolic characteristics. A slight, non-significant increase in HDL-C was noted, contrary to the reductions described in prior studies [3, 17], potentially influenced by patients’ physical activity levels or exclusive olive oil use. Finally, rather than the expected rise in uric acid typically associated with ketogenic diets, our study demonstrated a reduction (− 1.08 mg/dL, *p* = 0.014), which was greater than the decrease reported by Correa et al. [2] and opposite to the increase noted by Vinciguerra et al. [3], possibly attributable to better hydration practices.

### Effect of TRIF on Clinical Outcomes

Although postoperative weight loss following MBS is commonly attributed to reductions in energy intake, recent evidence suggests that the timing of food intake may also play a meaningful role. While no studies have evaluated the effects of intermittent fasting on RWG or SCR after MBS, limited research has examined the association between meal timing and postoperative weight outcomes [54–56]. In our study, the significant improvements observed in anthropometric parameters support the potential beneficial effects of the 16:8 time-restricted intermittent fasting (TRIF) regimen in individuals with obesity [56, 57]. Furthermore, our findings are consistent with previous studies reporting that intermittent fasting improves glucose and lipid metabolism [57–60].

### Comparative Effects of CBS, VLCKD, TRIF, and Control Groups on Clinical Outcomes

In our study, when comparing the groups, only a small difference in weight loss was observed between CBS and VLCKD; however, VLCKD produced greater weight reduction than CBS, TRIF, and the control group. While reductions in waist and hip circumference were more pronounced in the VLCKD group, the CBS group exhibited greater decreases in MUAC, fat mass, and muscle mass. All intergroup differences were statistically significant. The similar weight loss observed between CBS and VLCKD may be attributed to their comparable daily energy intake (692.9 kcal vs. 740 kcal). Despite this similarity, the CBS group experienced greater muscle mass loss, likely due to the higher protein intake in the VLCKD group (71.6 g vs. 57.0 g) and the lower physical activity levels typically observed during the early postoperative period.

When biochemical parameters were examined, improvements in glycemic indices and lipid profiles were observed in the CBS, VLCKD, and TRIF groups, whereas the control group showed minimal and heterogeneous changes. VLCKD produced the most pronounced improvement in glycemic markers, while CBS yielded more favorable alterations in lipid parameters. Although weight loss was comparable between VLCKD and CBS, the superior glycemic response in the VLCKD group may be attributable to the reduction in carbohydrate intake, which decreases insulin secretion and enhances insulin sensitivity. Supporting this mechanism, ketone bodies have been shown to positively modulate cellular insulin signaling [61]. Interestingly, despite achieving less total weight loss, TRIF resulted in a greater decrease in fasting insulin levels compared with CBS. This finding may be related to the higher frequency of insulin secretion triggered by the six-meal eating pattern adopted after CBS.

The more pronounced improvement in the lipid profile observed in the CBS group may be attributable to the lower total daily fat intake in these patients. Moreover, previous studies have demonstrated that performing RYGB as a CBS procedure enhances GLP-1 and PYY secretion and alters bile acid metabolism, mechanisms that strengthen hepatic cholesterol homeostasis and LDL receptor activity, thereby reducing plasma LDL-C levels [62]. Although TRIF resulted in less weight loss compared with VLCKD, it produced a more substantial improvement in lipid parameters, likely related to reduced insulin levels during the 16-hour fasting period, leading to increased lipolysis and suppression of hepatic VLDL production [63]. Collectively, these findings suggest that VLCKD and TRIF, despite being less potent than surgical procedures and associated with lower complication risks, may serve as viable non-surgical alternatives to CBS in the management of post-BS metabolic outcomes [64].

In our study, the CBS group showed increased consumption of milk, yogurt, and fruit, alongside reductions in refined carbohydrates, sweets, and sugary beverages, while the VLCKD and TRIF groups demonstrated higher intake of protein- and vegetable-based foods with concurrent decreases in snacks and high-energy items. These patterns indicate that all three interventions exerted favorable effects on dietary behavior, whereas improvements in the control group were minimal. Overall, the positive shifts observed in the intervention groups suggest that the structured nutrition plans and regular follow-up provided by the dietitian contributed to healthier dietary patterns.

Previous studies have shown that dietary patterns shift in patients experiencing RWG or SCR after MBS [65–68]. Chou et al. [66] reported that five years after SG, patients with RWG consumed lower total energy but relatively higher protein. In contrast, our cohort exhibited higher pre-intervention energy intake, along with lower protein and higher carbohydrate and fat intake. Similarly, Iossa et al. [65] reported greater total energy and carbohydrate/fat intake in RWG patients, whereas our control group showed comparable values.

Guidelines recommend ≥ 60 g/day protein and a macronutrient distribution of approximately 45% carbohydrate and 20–35% fat [16, 22, 69, 70, 71]. In our study, protein intake in the CBS, VLCKD, and control groups met these targets, whereas the TRIF group fell below recommendations. Across all groups, carbohydrate intake was lower and fat intake higher than guideline ranges; fiber intake was also below recommended levels. Overall, these findings indicate that RWG and SCR after SG may be influenced not only by total energy intake but also by macronutrient distribution, dietary quality, meal regularity, metabolic variation, and lifestyle factors.

Regular postoperative follow-up and dietitian-led counseling are known to reduce energy intake, improve dietary behaviors, and support sustained weight management after bariatric surgery [21, 72]. In our study, the greater improvements in anthropometric, biochemical, and nutritional outcomes in the CBS, VLCKD, and TRIF groups compared with the control group likely reflect both the interventions and the individualized nutrition plans with structured dietitian follow-up. These results highlight the role of continuous dietitian involvement in improving clinical outcomes and dietary habits.

This study represents the first randomized controlled trial comparing CBS, VLCKD, and TRIF in patients who experienced SCR or RWG following bariatric surgery, thereby contributing to the existing literature. Furthermore, the absence of prior research examining the impact of TRIF on anthropometric and biochemical parameters, as well as dietary habits, in this patient population enhances the originality and potential clinical relevance of our findings. In this context, the study is noteworthy for evaluating both surgical (CBS) and non-surgical nutritional interventions within a comparative framework.

This study has several limitations. First, the relatively small sample size may reduce statistical power and limit generalizability. Additionally, the six-week intervention reflects only short-term outcomes; therefore, the long-term sustainability of weight loss and associated metabolic and behavioral changes remains uncertain. Larger studies with extended follow-up are warranted. Dietary intake was assessed using self-reported records, which may introduce reporting bias. Finally, patients with SCR and RWG were not analyzed separately; future studies should consider stratified analyses, as these groups may exhibit distinct pathophysiological and behavioral characteristics.

## Conclusions

This study demonstrates that, in patients who experienced SCR or RWG following bariatric surgery, CBS, VLCKD, and TRIF resulted in significant short-term improvements in anthropometric outcomes, metabolic parameters, and dietary habits. While VLCKD and TRIF appear to be feasible non-surgical alternatives, their achievement of outcomes comparable to CBS is clinically relevant. Nevertheless, further studies with larger sample sizes and longer follow-up periods are needed to assess long-term efficacy and sustainability. Collectively, these findings suggest that in post-bariatric surgery weight management, not only surgical interventions but also structured, nutrition-based strategies—implemented under dietitian supervision and monitored by a multidisciplinary team—may represent effective and feasible treatment options.

## Supplementary Information

Below is the link to the electronic supplementary material.

Supplementary Material 1.

## Data Availability

The datasets generated and analyzed during the current study are not publicly available due to privacy restrictions but are available from the corresponding author on reasonable request.
