# Supplementary material for: Conversion Bariatric Surgery, Ketogenic Diet, and Intermittent Fasting in Bariatric Surgery Patients with Recurrent Weight Gain: a Prospective Randomized Controlled Trial
Source: Obes Surg. 2026 Apr 11;36(6):2857–68. doi: 10.1007/s11695-026-08654-w (PMC13249736; doi:10.1007/s11695-026-08654-w)
Supplement: Supplementary file 1 — Supplementary Material 1. [file 11695_2026_8654_MOESM1_ESM.docx]

**Supplementary Tables**

**Supplementary Table 1. Dietary habits of patients in the CBS group before and after the intervention**

| Dietary items | Comparison | p |
| --- | --- | --- |
| Milk | Pre-Post | 0.117 |
| Yogurt | Pre-Post | 0.490 |
| Cheese | Pre-Post | 0.026 |
| Egg | Pre-Post | 0.032 |
| Meat/Chicken/Fish | Pre-Post | <0.001 |
| Legumes | Pre-Post | 0.016 |
| Bread | Pre-Post | 0.621 |
| Bulgur wheat/rice /pasta/potato | Pre-Post | 0.176 |
| Pastry products | Pre-Post | 0.285 |
| Vegetables | Pre-Post | 0.042 |
| Salad | Pre-Post | 0.139 |
| Fruit | Pre-Post | 0.230 |
| Butter | Pre-Post | 0.364 |
| Margarine | Pre-Post | 0.029 |
| Olive oil | Pre-Post | 0.038 |
| Sunflower/corn oil | Pre-Post | 0.001 |
| Oilseed | Pre-Post | 0.049 |
| Chips | Pre-Post | 0.156 |
| Cup of coffee | Pre-Post | 0.490 |
| Soft drink | Pre-Post | 0.175 |
| Carton fruit juice | Pre-Post | 0.429 |
| Fresh fruit juice | Pre-Post | 0.146 |
| Alcohol | Pre-Post | 1.000 |
| Milky dessert | Pre-Post | 0.899 |
| Syrupy dessert | Pre-Post | 0.880 |
| Chocolate | Pre-Post | 0.885 |
| Biscuit, cracker etc. | Pre-Post | 0.880 |

p= Bowker test

**Supplementary Table 2. Dietary habits of patients in the VLCKD group before and after the intervention**

| Dietary items | Comparison | p |
| --- | --- | --- |
| Cheese | Pre-Post | <0.001 |
| Egg | Pre-Post | 0.066 |
| Meat/Chicken/Fish | Pre-Post | 0.845 |
| Vegetables | Pre-Post | 0.156 |
| Salad | Pre-Post | 0.821 |
| Butter | Pre-Post | 0.897 |
| Olive oil | Pre-Post | 0.010 |
| Sunflower/corn oil | Pre-Post | 0.093 |
| Oilseed | Pre-Post | 0.725 |
| Chips | Pre-Post | 1.000 |
| Cup of tea | Pre-Post | 0.008 |
| Cup of coffee | Pre-Post | 0.006 |
| Soft drink | Pre-Post | 1.000 |
| Fresh fruit juice | Pre-Post | 1.000 |
| Alcohol | Pre-Post | 0.148 |

Milk, yogurt, legumes, bread, bulgur wheat/rice/pasta/potato, pastry products, fruit, margarine, carton fruit juice, milky desserts, syrupy desserts, chocolate, and biscuits/crackers showed complete agreement; therefore, Bowker’s test of symmetry could not be computed because the classifications were identical across pre-post observations (perfect agreement).

**Supplementary Table 3. Dietary habits of patients in the TRIF group before and after the intervention**

| Dietary items | Comparison | p |
| --- | --- | --- |
| Milk | Pre-Post | 0.001 |
| Yogurt | Pre-Post | 0.001 |
| Cheese | Pre-Post | 0.184 |
| Egg | Pre-Post | 0.030 |
| Meat/Chicken/Fish | Pre-Post | 0.116 |
| Legumes | Pre-Post | 0.097 |
| Bread | Pre-Post | 0.061 |
| Bulgur wheat/rice /pasta/potato | Pre-Post | <0.001 |
| Pastry products | Pre-Post | 0.783 |
| Vegetables | Pre-Post | <0.001 |
| Salad | Pre-Post | 0.033 |
| Fruit | Pre-Post | 0.076 |
| Butter | Pre-Post | 0.015 |
| Margarine | Pre-Post | 0.080 |
| Olive oil | Pre-Post | 0.002 |
| Sunflower/corn oil | Pre-Post | 0.032 |
| Oilseed | Pre-Post | <0.001 |
| Chips | Pre-Post | 0.156 |
| Cup of tea | Pre-Post | <0.001 |
| Cup of coffee | Pre-Post | <0.001 |
| Soft drink | Pre-Post | 0.215 |
| Carton fruit juice | Pre-Post | 0.074 |
| Fresh fruit juice | Pre-Post | 0.001 |
| Alcohol | Pre-Post | <0.001 |
| Milky dessert | Pre-Post | 0.401 |
| Syrupy dessert | Pre-Post | 0.084 |
| Chocolate | Pre-Post | 0.537 |
| Biscuit, cracker etc. | Pre-Post | 0.169 |

p= Bowker test

**Supplementary Table 4. Dietary habits of patients in the Control group before and after the intervention**

| Dietary items | Comparison | P |
| --- | --- | --- |
| Milk | Pre-Post | <0.001 |
| Yogurt | Pre-Post | 0.016 |
| Cheese | Pre-Post | 1 |
| Egg | Pre-Post | 0.338 |
| Legumes | Pre-Post | 0.001 |
| Bread | Pre-Post | 0.003 |
| Vegetables | Pre-Post | 0.001 |
| Salad | Pre-Post | 0.010 |
| Fruit | Pre-Post | 0.001 |
| Butter | Pre-Post | 0.023 |
| Chips | Pre-Post | 0.004 |
| Cup of coffee | Pre-Post | 1,000 |
| Soft drink | Pre-Post | 0.039 |
| Carton fruit juice | Pre-Post | 0.163 |
| Milky dessert | Pre-Post | 0.056 |
| Syrupy dessert | Pre-Post | 0.008 |
| Biscuit, cracker etc. | Pre-Post | 0.819 |

Meat/chicken/fish, bulgur wheat/rice/pasta/potato, pastry products, margarine, olive oil, sunflower/corn oil, oilseeds, cups of tea, fresh fruit juice, alcohol, and chocolate showed complete agreement; therefore, Bowker’s test of symmetry could not be computed because the classifications were identical across pre-post observations (perfect agreement).
